# Editorial: Transforming food by-products into functional ingredients for sustainable nutrition

**DOI:** 10.3389/fnut.2026.2016793

**Published:** 2026-10-28

**Authors:** José Pinela, Sandrina A. Heleno

**Affiliations:** 1 Instituto Nacional de Investigação Agrária e Veterinária (INIAV, I.P.), Vila do Conde, Portugal; 2 CIMO, LA SusTEC, Universidade Politécnica de Bragança, Campus de Santa Apolónia, Bragança, Portugal

**Keywords:** bioactive compounds, bioavailability, by-product valorization, extraction methods, food processing, functional foods, novel food formulation, upcycled food ingredients

The development of sustainable food systems increasingly depends on identifying new applications for biomaterials currently underutilized or discarded as waste. Food production and processing stages generate large quantities of by-products that may be rich in nutrients, bioactive compounds, and other constituents with nutritional, functional, or technological value. However, upcycling these resources into food-grade ingredients requires more than simply characterizing their chemical composition or demonstrating their bioactive properties *in vitro*. Their valorization requires appropriate processing to unlock their technological and functional potential, as well as evidence of safety, techno-economic feasibility, and consumer acceptance. The contributions gathered in this Research Topic of Frontiers in Nutrition reflect this multidisciplinary approach, encompassing the processing and characterization of plant- and animal-derived biomass for the development of bio-based ingredients, the evaluation of their functional properties and biological effects, and their incorporation into food products (Figure 1).

**Figure 1:**
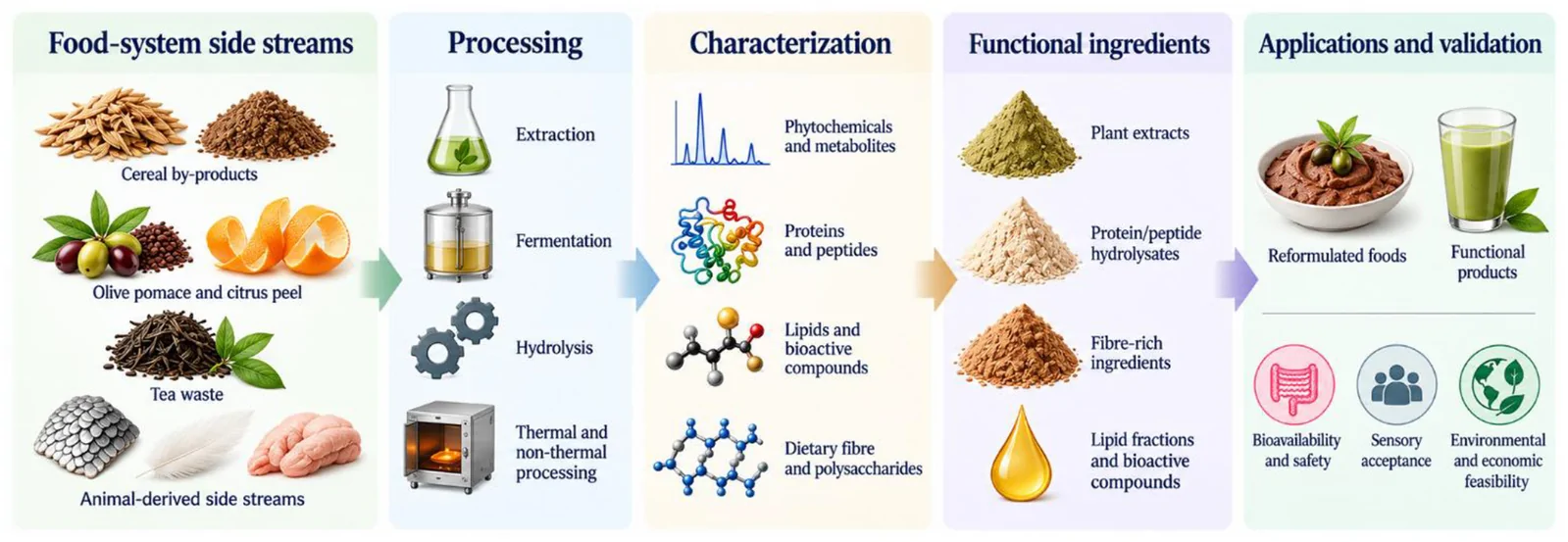
Valorization pathways addressed in this Research Topic for plant- and animal-derived by-products, from their processing and characterization to functional ingredient development and food applications.

The diversity of underutilized resources that may be valorized is reflected in the studies included in this Research Topic. Nut-processing by-products, including shells, skins, and other residual fractions, were investigated by Zhou et al. as possible sources of polysaccharides, phenolic compounds, and other functional constituents. In addition to their antioxidant, antimicrobial, and anti-inflammatory properties and potential applications in food products, challenges that limit the upcycling of these by-products have been highlighted, including compositional variability, limited extraction efficiency, and insufficient data on bioavailability, metabolite transformation, and long-term safety of derived extracts. The valorization potential of camel hump fat, an underused livestock by-product rich in saturated (63.4%) and monounsaturated (34.7%) fatty acids, was discussed by Yasen et al. Opportunities for its valorization in food, cosmetic, and biomaterial industries were highlighted, as well as the need for additional studies on safety and functionality assessment. Yuan et al. explored the skin of farm-raised giant salamanders as a source of eumelanin and evaluated its metabolism and hemostatic effects *in vivo*. Following administration of the extracted eumelanin to male Wistar rats, antifibrinolytic metabolites were identified in the intestinal metabolome, together with changes in several amino acids. Eumelanin also reduced hemorrhage volume and hemostasis time without altering coagulation parameters beyond physiological ranges, supporting the valorization of this animal by-product. Regarding medicinal-food homologous plants, Chen et al. have reviewed their potential for the prevention and management of hepatic fibrosis, highlighting mechanisms involving oxidative stress and inflammation, hepatic stellate cell activation, and the gut-liver axis. Translational gaps, including the frequent use of poorly characterized extracts and limited pharmacokinetic, toxicological, and clinical evidence, were also pointed out by the authors. Overall, these studies illustrate the diverse nature of by-products with potential for valorization and highlight the importance of moving beyond chemical characterization to assess their functional and biological relevance.

Other studies demonstrate how processing can modify the properties of underutilized side streams. Jun et al. investigated black rice bran, a rice-milling by-product, after fermentation with *Lentinula edodes*. The water-soluble fraction obtained from the fermented product reduced fat accumulation in *Caenorhabditis elegans* and attenuated age-related declines in motility and intestinal barrier integrity, supporting the potential of fermented black rice bran as a functional food ingredient. Aldayel showed that He-Ne laser irradiation can modulate curcuminoid accumulation in *Curcuma longa* rhizomes and enhance antioxidant and antimicrobial activities, thereby illustrating how this non-thermal, chemical-free approach may be used to modify the functional properties of plant-derived ingredients. The importance of processing was also evident in the study of brewer's spent grain by Minestrini et al. LC-MS/MS analysis showed that oven-drying significantly affected the profiles of sugars, amino acids, and phenolic compounds, whereas the extraction solvent was particularly influential for lipids and hordatines. The highest antioxidant capacity was observed in extracts prepared from unprocessed/fresh material, particularly using 40% ethanol, suggesting that drying and stabilization may also affect desired quality attributes. Xu et al. also demonstrated the importance of the extraction method by comparing steam distillation and supercritical fluid extraction (SFE) for the recovery of essential oil from zhoupigan (*Citrus reticulata* cv. Manau Gan) peels. The obtained oils presented distinct volatile and aroma profiles and differences in *in vitro* antioxidant and antibacterial activities, with SFE showing better performance.

The importance of processing for the valorization of carp scales, whose rigid structure and low nutrient bioavailability limit their direct use, was demonstrated by Yan et al. Sand stir-frying (150 °C, 2.5 min) improved their nutritional profile by increasing protein content and generating novel low-molecular-weight peptides. These biochemical changes translated into greater anti-inflammatory and organ-protective efficacy in a mouse model of lipopolysaccharide-induced sepsis compared with raw scales. In turn, a keratin hydrolysate derived from poultry feathers was investigated by Wauquier et al., who used serum collected from healthy volunteers after ingestion of the hydrolysate. Glycosaminoglycan and hyaluronan production and early migration in primary human dermal fibroblasts were promoted by the post-ingestion serum, while markers of oxidative stress, senescence, and apoptosis were reduced. Despite these promising findings for the valorization of aquaculture and poultry industry by-products, further studies are needed to elucidate the underlying mechanisms and determine whether these effects translate into clinically relevant benefits in humans.

Regarding direct applications of by-products in food, Melo Ferreira et al. incorporated pasteurized olive pomace paste into high-pressure-processed olive pâtés. The enriched formulations contained higher levels of dietary fiber and vitamin E and showed higher antioxidant capacity than a commercial olive pâté. Additionally, while hydroxytyrosol was not detected in the commercial product, significant amounts were quantified in the fortified pâtés, which also had good consumer acceptance, particularly the formulation with 20% olive pomace paste. Cifte et al., on the other hand, converted black tea waste fiber into instant tea powders using hot (100 °C, 6 min) and cold (25 °C, 2 h) extractions. Hot extraction yielded higher levels of catechins, condensed phenolics, and theanine and showed antiproliferative effects on prostate cancer cells and strong cytotoxicity against U87MG glioblastoma cells. Cold extraction resulted in higher levels of gallic acid and caffeine and exhibited stronger inhibitory effects on MCF7 breast cancer cells. The potential of food processing by-products for upcycling into functional foods was demonstrated by these two studies, which highlighted the need to consider consumer acceptance, bioavailability, and biological effects during the valorization process.

In conclusion, the studies in this Research Topic demonstrate that the successful valorization of food by-products requires more than chemical characterization. Their processing must be tailored to their physicochemical properties and intended application, as different approaches can either enhance or compromise desirable attributes. Similarly, chemical composition alone does not guarantee functionality, and promising *in vitro* results must, where relevant, be supported by evidence of bioaccessibility and bioavailability, safety, and relevance to human health and nutrition. Sustainability must also be demonstrated, not assumed, especially when valorization pathways involve resource-intensive extraction or processing steps, making scalability, environmental impact, and economic feasibility important considerations. Therefore, the challenge is not simply to find new applications for food by-products and processing side streams, but to sustainably transform them into safe, effective, acceptable food ingredients. In this transition from by-product to validated bio-based food ingredient, sustainability and nutrition must be considered together.

